# Characterization of an *Enterococcus faecalis* Bacteriophage vB_EfaM_LG1 and Its Synergistic Effect With Antibiotic

**DOI:** 10.3389/fcimb.2021.698807

**Published:** 2021-07-16

**Authors:** Min Song, Dongmei Wu, Yang Hu, Haiyan Luo, Gongbo Li

**Affiliations:** Department of Neurology, The Second Affiliated Hospital of Chongqing Medical University, Chongqing, China

**Keywords:** bacteriophage, phage-antibiotic combination, *Enterococcus faecalis*, antibiotic resistance, phage therapy

## Abstract

*Enterococcus faecalis* is a Gram-positive opportunistic pathogen that could cause pneumonia and bacteremia in stroke patients. The development of antibiotic resistance in hospital-associated *E. faecalis* is a formidable public health threat. Bacteriophage therapy is a renewed solution to treat antibiotic-resistant bacterial infections. However, bacteria can acquire phage resistance quite quickly, which is a significant barrier to phage therapy. Here, we characterized a lytic *E. faecalis* bacteriophage Vb_EfaM_LG1 with lytic activity. Its genome did not contain antibiotic resistance or virulence genes. Vb_EfaM_LG1 effectively inhibits *E. faecalis* growth for a short period, and phage resistance developed within hours. However, the combination of antibiotics and phage has a tremendous synergistic effect against *E. faecalis*, prevents the development of phage resistance, and disrupts the biofilm efficiently. Our results show that the phage-antibiotic combination has better killing efficiency against *E. faecalis*.

## Introduction

*Enterococci* are Gram-positive facultative anaerobes, examples of which include *E. faecalis* and *Enterococcus faecium*, which cause bacteremia, pneumonia, endocarditis, and urinary tract infections (Beganovic et al., 2018; Jabbari Shiadeh et al., 2019). In addition, *E. faecalis* is also one of the major pathogens for pneumonia and bacteremia in stroke patients, and the infection after stroke could lead to the death of the stroke patient (Hannawi et al., 2013; Dyal and Sehgal, 2015; Stanley et al., 2016). Moreover, the intrinsic and acquired antibiotic resistance of *Enterococci* is a formidable public health threat (Arias et al., 2011; Banla et al., 2018). *Enterococci* have evolved extensive drug resistance, including that to vancomycin, and could transmit antibiotic resistance among diverse bacteria (Palmer et al., 2010). Therefore, new therapeutic approaches are needed to treat *Enterococcal-*associated infections (Khalifa et al., 2015; Kortright et al., 2019; Bao et al., 2020).

Phages are viruses that infect and kill bacteria and are used to treat antibiotic-resistant bacteria (Barbu et al., 2016; Kortright et al., 2019; Dion et al., 2020). Phage therapy has several advantages over antibiotics. First, phages have a particular host range and only infect the targeted bacterium, so phage therapy would not affect other bacteria and did not interrupt the commensal microbes (Waters et al., 2017). Second, because of the different phage resistance and antibiotic resistance mechanisms in bacteria, phages could infect multidrug-resistant superbugs. Thus, phage therapy is being proceeded in many countries (Jault et al., 2018; Leitner et al., 2021).

Currently, numerous phages against pathogens had been characterized; however, there are only 63 sequenced *E. faecalis* bacteriophage deposited in NCBI (Chatterjee et al., 2021), which is relatively understudied compared with phages that infect other pathogens, such as *Pseudomonas aeruginosa* or *Staphylococcus aureus* phages (De Smet et al., 2017). More phages need to be characterized to provide more therapeutic options for treating the multidrug-resistant *E. faecalis*. Moreover, phage resistance is quite common for *E. faecalis*, which could be quickly selected because of the mutations of cell wall-associated polysaccharide or membrane protein (Duerkop et al., 2016; Banla et al., 2018; Chatterjee et al., 2019). Thus, a better strategy to hinder phage resistance should be investigated. In this study, we identified a phage infecting a broad range of *E. faecalis* strains and proved that phage-antibiotic synergism effectively inhibits phage resistance and disrupts biofilm.

## Results

### The Biology of an *E. faecalis* Phage

**Figure 1:**
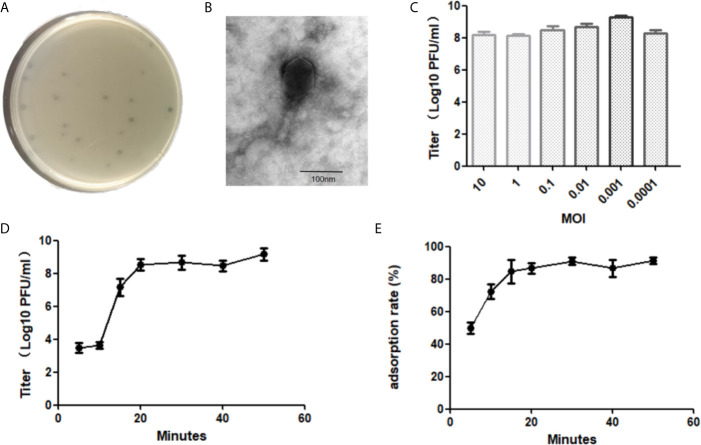
Biological characterization of *E. faecalis* phage vB_EfaM_LG1. The plaque **(A)** and transmission electron micrograph **(B)** of LG1. **(C)** The optimal MOI test of phage. **(D)** The one-step growth curve of LG1. **(E)** The adsorption rate of LG1 against host strain ef118 within 60 min.

A phage was isolated from the hospital sewage using *E. faecalis* strain ef118 as a host. It forms an obvious plaque on the host in the double layer agar plates (**Figure 1A**). The phage particle was extracted from the bacterial lysate and was observed by transmission electron microscopy. The head of the phage is a regular icosahedral structure with a diameter of approximately 80 nm, and it has a contractable tail with a length of approximately 110 nm (**Figure 1B**). Thus, the morphology of this phage conforms to the characteristics of the Myoviridae family, and it is named *Enterococcus faecalis* phage vB_EfaM_LG1 (refer as LG1 hereafter).

The phage titer reached the highest as 4 × 10^9^ PFU/ml when the multiplicity of infection (MOI) was 0.001, the optimal MO of bacteriophage LG1 was 0.001 (**Figure 1C**). The one-step growth curve of LG1 was shown in **Figure 1D**. The latent phase was approximately 10 min, and then the titer of phages increased rapidly between 10 and 20 min, indicating a lysis period of approximately 20 min. The burst size was estimated as about 40 pfu per bacterium.

The adsorption rate of LG1 onto the host strain was determined by measuring the remaining phages in the supernatant. LG1 absorbed onto the host ef118 efficiently, and over 50% of the phage particles were adsorbed by the ef118 within 5 min, and approximately 80% of the phage could bind to the host within 20 min (**Figure 1E**).

Spot agar assays were performed to determine the phage infectivity against 10 *E. faecalis* clinically isolated strains. The formation of clear plaques indicates that the strain is sensitive to LG1, whereas the formation of blurred plaque or no spots is considered non-sensitive. LG1 infects 50% of the clinical isolated *E. faecalis* strains, representing a relatively broad host range, but LG1 cannot infect any *E. faecium* strain (**Table 1**).

**Table 1:** The host range of phage LG1.

Strain	Origin	LG1 sensitivity
*Enterococcus faecalis* ef118	Blood	+
*Enterococcus faecalis* ef122	Blood	−
*Enterococcus faecalis* ef153	Blood	−
*Enterococcus faecalis* ef177	Blood	+
*Enterococcus faecalis* ef134	Blood	+
*Enterococcus faecalis* ef189	Urine	−
*Enterococcus faecalis* ef101	Urine	+
*Enterococcus faecalis* ef116	Urine	−
*Enterococcus faecalis* ef126	Urine	+
*Enterococcus faecalis* ef148	Urine	−
*Enterococcus faecium* ef13	Blood	−
*Enterococcus faecium* ef14	Urine	−
*Enterococcus faecium* ef15	Blood	−
*Enterococcus faecium* ef16	Urine	−

+ indicates the strain is sensitive to phage LG1 and forms clear plaque; − indicates the strain is not sensitive to phage LG1.

### Sequencing Analysis of an *E. faecalis* Phage LG1

Phage LG1 is a double-stranded (ds) DNA phage with a linear genome of 150,025 base pairs (bp). Its G + C content is 35.88%, and the genome is visualized by CPT Phage Galaxy (Ramsey et al., 2020).

**Figure 2:**
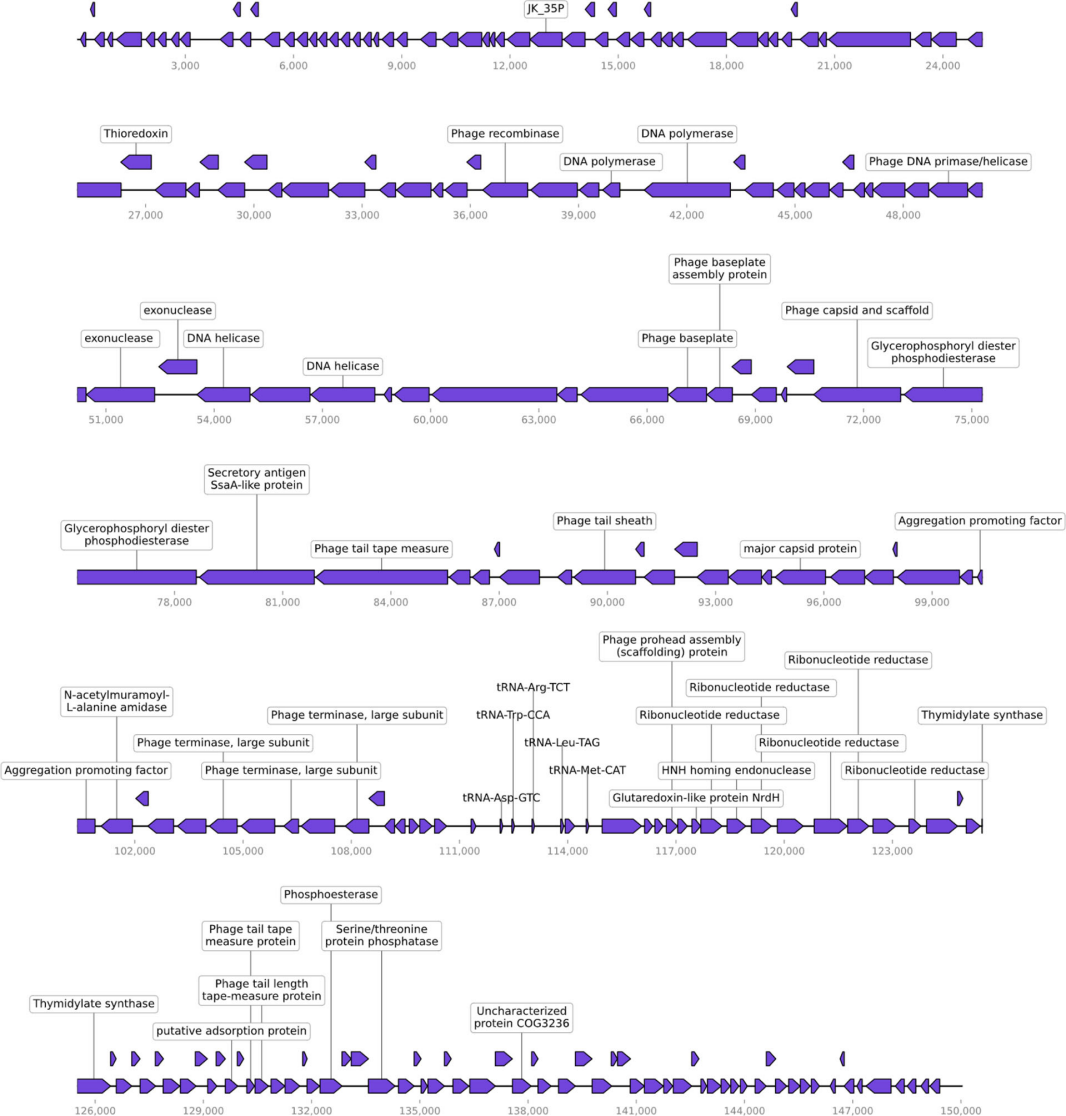
Genomic characterization of vB_EfaM_LG1. LG1 is a dsDNA phage with 231 proteins predicted based on sequence homology and five tRNA genes.

There are 231 putative ORFs predicted by RAST (Overbeek et al., 2014), whereas most of the ORFs are functionally unknown. Also, LG1 encodes five tRNA genes. The annotated ORFs can be categorized into several functional modules, including phage replications, DNA metabolism/modifications, lysis, phage structural protein (**Figure 2**). LG1 encodes multiple ribonucleotide reductases, implying that LG1 could perform *de novo* DNA biosynthesis. Moreover, no antibiotic-resistant genes or virulence genes were predicted in the genome of LG1. BlastN searches of the non-redundant database at NCBI reveals that LG1 genome exhibits 90% to 98% nucleotide identity with a group of enterococcal phages, such as *Enterococcus* phage ECP3 and vB_EfaM_Ef2.1.

### Stability of LG1

**Figure 3:**
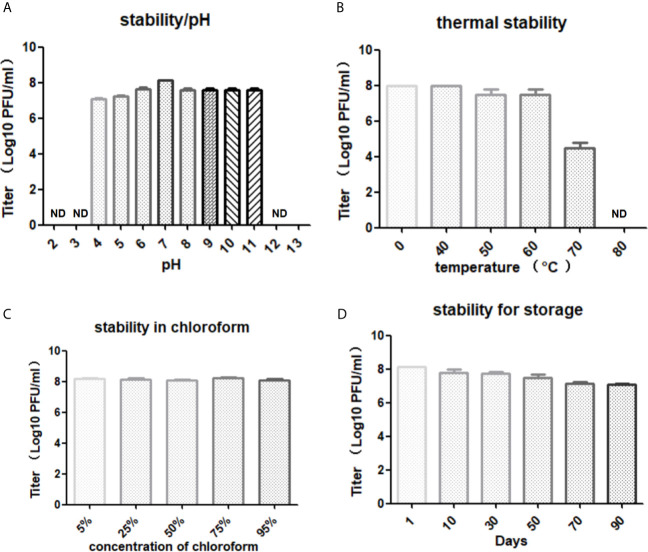
Stability of LG1. **(A)** Phage LG1 is stable under pH4~11 but significantly inactivated under pH4 or above pH11. **(B)** LG1 is inactivated by 80°C treatment. **(C)** LG1 is non-sensitive to chloroform treatment. **(D)** LG1 is stable for 3 months without a significant decrease of titer when stored at 4°C. ND, not detected.

The optimal pH for storing LG1 was 7, and its viability was lost entirely when the pH was lower than 4 or higher than 11 (**Figure 3A**). The phage titers were further monitored when LG1 was incubated at different temperatures. It was found to be stable at different temperatures, maintained a titer of 10^5^ after 60 min incubation at 70°C (**Figure 3B**), and was wholly inactivated over 80°C. Besides, chloroform treatment did not affect the phage titer, precluding the presence of lipid components on the phage surface (**Figure 3C**). Finally, the chloroform-treated phage was stored at 4°C, and its titer was monitored for 3 months (**Figure 3D**). And the titer of LG1 did not significantly decrease during this period, indicating LG1 was relatively stable at 4°C, and this feature is vital to produce phage agents.

### The Phage-Antibiotic Combination Significantly Inhibits the Development of Phage Resistance and Disrupts the Biofilm

**Figure 4:**
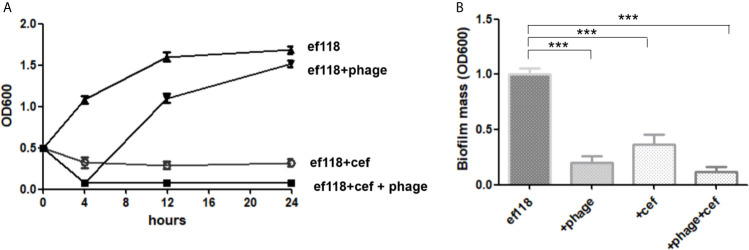
Phage-antibiotic synergism. **(A)** Phage could only inhibit the growth of *E. faecalis* for several hours, and then the phage resistance mutant grows to a high density. **(B)** The phage-antibiotic combination has better efficacy in destroying the biofilm than phage or antibiotic alone (***P < 0.05).

Phage resistance is quickly developed and is selected *in vitro* and *in vivo* (Labrie et al., 2010). And phage resistance in *E. faecalis* can be achieved through mutations of the receptors on the cell surface (Duerkop et al., 2016). As expected, in the liquid culture, phage LG1 was added to the log phase ef118 (OD600 = 0.5) to a final titer of 5 × 10^8^ pfu/ml, and LG1 could only inhibit *E. faecalis* ef118 for several hours, and phage-resistant mutants regrow to a high density within 24 h (**Figure 4A**). The sensitive antibiotic cefotaxime (32 µg/ml) could inhibit the ef118, but the phage-antibiotic combination shows the best killing efficiency (**Figure 4A**). And in the *in vitro* biofilm model, cefotaxime (32 µg/ml) is less effective in disrupting the established biofilm than phage (5*10^8^ pfu/ml) alone, and the phage-antibiotic combination has a more significant effect in disrupting the biofilm than single treatment (**Figure 4B**).

## Discussion

With an ever-increasing amount of antibiotic-resistant strains of *E. faecalis* found in clinical and the difficulties in the treatment of those caused by the biofilm formation (Arias et al., 2011; Palmer et al., 2011). A better strategy to constrain *E. faecalis* infection is needed more than ever, and lytic bacteriophage is a promising alternative treatment to fight multidrug-resistant *E. faecalis* (Al-Zubidi et al., 2019). In this study, we isolated a dsDNA phage LG1, which effectively infects *E. faecalis* strains with a relatively broad host range. Transmission electron microscopy showed that the phage belongs to the Myoviridae family, and its genome sequence exhibited similarity to other *E. faecalis* phages in the Myoviridae family.

Phage stability is an essential parameter for manufacturing phage agents (Pires et al., 2020). In Phagoburn project, researchers found that the phage cocktail is significantly inactivated because of long-term storage, and the phage titer is as low as 10^2^ pfu/ml per daily dose, which is one of the reasons for the failure of this phage therapy clinical trial (Jault et al., 2018). LG1 is stable under different conditions, including heat and pH, and it can be stored at 4°C without significant loss of the titer for 3 months, which is an important parameter when LG1 is included in a phage cocktail agent.

Phage resistance is also an issue in phage therapy. Because bacteria are able to obtain phage resistance because of various mechanisms, including mutations of the receptor, restriction and modification systems, CRISPR-cas systems (Labrie et al., 2010; Goldfarb et al., 2015; Shen et al., 2018b; Azam and Tanji, 2019), and phage resistance have been reported in phage therapy cases (El Haddad et al., 2019; Bao et al., 2020), which is a severe issue in phage therapy. *E. faecalis* phage resistance has been investigated previously, mainly through the mutation of phage receptor, including membrane protein PIP for phage phiVPE25 (Duerkop et al., 2016) and enterococcal polysaccharide antigen for phage (Chatterjee et al., 2019). Various approaches had been suggested to inhibit the development of phage resistance. Phage-antibiotic is the well-acknowledged method in treating other pathogens, such as *P. aeruginosa* (Oechslin et al., 2017). This study also suggests that phage-antibiotic combination is a better strategy to treat *E. faecalis* infection.

Formation of biofilm is a severe issue in infections because the established biofilm is extremely difficult to disrupt, and the biofilm increases antibiotic resistance (Pires et al., 2017). Phage effectively disrupts biofilm because phage could penetrate the biofilm, and some phage encodes depolymerase to degrade the biofilm matrix to destroy further the biofilm. Depolymerases can be associated with the phage particle or be released during lysis of the host bacteria. Depolymerases are enzymes to degrade the extracellular polysaccharide. Therefore, it is particularly interesting in the removal of biofilms. (Wu et al., 2019; Ferriol-Gonzalez and Domingo-Calap, 2020).

Moreover, this experiment shows phage-antibiotic combination has better effects in treating biofilms, which would be a better approach to treat chronic *E. faecalis* when biofilm might have already formed. The phage-antibiotic synergism is mainly because of the different antibacterial targets. And under certain conditions, phages provide an adjuvating effect by lowering the minimum inhibitory concentration for drug-resistant strains to enhancing the effect of antibiotics (Liu et al., 2020). Overall, these data indicate phage-antibiotic synergism has better treating efficiency than single phage therapy.

### Experimental Procedures

#### Bacterial Strains, Phages, and Culture Conditions

The bacterial strains in this work were listed in **Table 1**. *Enterococcus* strains were collected from the Department of Clinical Laboratory Medicine and grown aerobically on Luria-Bertani (LB) broth at 37°C.

Bacteriophage LG1 was isolated from hospital sewage as previously described (Duerkop et al., 2016). Briefly, the sewage was pelleted, and the supernatant was filtered through a 0.45-µm pore-size filter to remove particles. Then, 50-µl sample was immediately mixed with 200 µl bacterial culture, and 4 ml of molten LB soft agar (0.7%) was added and poured onto LB agar plates, followed by overnight culture. Any formed plaque was picked using a pipette, deposited in 1 ml of LB, followed by 10-fold dilution, and double-layer agar assay to purify the phage. The phage was purified by three consecutive rounds. Then, one plaque from the third round was picked for this study.

#### Transmission Electron Microscopy

Phage particles were dropped on carbon-coated copper grids for 10 min. Then phosphotungstic acid (pH 7.0) was used to stain the sample for 15 s and examined under a Philips EM 300 electron microscope. The sizes of the phage were measured based on five randomly selected images using AxioVision LE.

#### Phage Titering and MOI Experiment

Phage titer was calculated by standard double-layer agar plate assay. Briefly, 10-fold dilutions of phage suspension were mixed with 200 µl host bacteria, then mixed with 5 ml molten 0.7% LB agar broth. Then poured on a 1.5% agar plate. After overnight incubation at 37°C, one plaque is calculated as a plaque-forming unit (pfu). MOI experiments were performed by mixing log-phase bacteria (OD600 = 0.5) with a different number of phages, and the coculture was incubated at 37°C with shaking for 5 h. Then the titer in the supernatant was calculated using a double-layer agar plate assay.

#### One-Step Growth

The one-step growth curve of LG1 was determined as described (Zhong et al., 2020). Briefly, 1 ml of log-phase bacteria and 1 ml of LG1 were mixed at an MOI of 1 and incubated at 37°C for 3 min. Then, the mixture was centrifuged at 4°C for 2min at a speed of 12,000*g*, and the pellet was resuspended in 10 ml LB medium. And samples were taken at the given time points, which are immediately pelleted, and phage titer in the supernatant was measured by directly using double-layer plate assay.

### Adsorption Rate Experiments

Bacteriophage adsorption assay with various time points was performed as previously described (Al-Zubidi et al., 2019). Briefly, the log phase bacterial cultures were pelleted and resuspended in LB medium to a final concentration of 5 × 10^8^ CFU/ml. Then, phage was added to a final titer of 5 × 10^5^ pfu/ml. Then, the samples were cultured at 37°C for 60 min, and a 1-ml sample was collected at the set time point and centrifuged at 16,000*g* for 1 min. The phages in the supernatant were titered using the double-agar plating assays. At a given time point, the adsorption rate was calculated as (the original phage titer − the remaining phage titer)/the original phage titer.

#### Determination of Host Range

Ten *E. faecalis* and five *E. faecium* strains were selected as test strains. The host range of phage LG1 was determined using spot testing by dropping 1 µl of phage onto the double-layer soft agar premixed with the test strain and cultured at 37°C for 18 h. The formation of a clear plaque is considered as the sensitive host for phage LG1.

#### Isolation of Bacteriophage DNA

The phage DNA extraction is performed as previously described (Khan et al., 2021). Briefly, DNase I and RNase A were added to a final concentration of 5 and 1 μg/ml, respectively, and the purified phage particle was treated for 1 h at 37°C. Proteinase K (final concentration of 50 μg/ml), EDTA (pH 8.0), and 0.5% SDS were added and treated at 56°C for 1 h. Then, phage genome DNA was extracted with saturated phenol (pH 8.0). After centrifugation, the aqueous phase was extracted with chloroform and mixed with the same volume of isopropyl alcohol and stored at −20°C for 1 h. Then, phage DNA was precipitated by centrifugation and was washed with 70% ethanol and absolute ethanol, respectively. After drying, the precipitate was dissolved in TE solution, and the phage DNA was stored at −80°C.

#### Genome Sequencing and Annotation

Phage genomic DNA was sequenced using an Illumina Hiseq 2500 platform (~1 Gbp/sample). Fastp (Chen et al., 2018) was used for adapter trimming and quality filtering after demultiplexing the raw reads. The read data were assembled using the *de novo* assembly algorithm Newbler Version2.9 with default parameters, and the assembled genome was annotated by RAST. The DNA and protein sequences were checked for homologs with BLAST manually. The genome map was drawn by a phage genome visualization online software CPT Phage Galaxy (Ramsey et al., 2020). The sequence data are available in the NCBI under accession number MZ420150.

#### Stability Studies

To test the phage stability under various conditions, 10^8^ pfu of LG1 was treated with different pH, temperature, or chloroform for 60 min, then the titer of the phage was calculated by double-layer agar assay. The LG1 was stored at 4°C, and its titer was determined at the given time points for 3 months.

#### Biofilm Assay

Biofilms were examined by the crystal violet staining method as previously described (Shen et al., 2018a). Briefly, 0.2 ml of log-phase bacterial culture were added to 96-well polystyrene microplates and incubated for 24 h at 37°C to establish biofilm. Then, the untreated control wells were washed with phosphate-buffered saline (PBS) and stained with crystal violet for 15 min, which was solubilized in 0.2 ml of 95% ethanol, and the biofilm biomass was estimated by measuring the OD 600, which was determined using a SpectraMax M3 multimode microplate reader. For the treatment groups, the wells were washed and PBS, then 0.2 ml of phage or antibiotic was added and incubated at 37°C for 4 h, the biofilm biomass was determined by crystal violet staining method.

#### Statistical Analysis

All the experiments were performed three times, and statistical analysis was performed using one-way ANOVA or t-test, and statistical significance was assumed if the P value was <0.05.

## Data Availability Statement

The data sets presented in this study can be found in online repositories. The names of the repository/repositories and accession number(s) can be found below: https://www.ncbi.nlm.nih.gov/, MZ420150, PRJNA723057.

## Author Contributions

All the authors listed have made a substantial, direct and intellectual contribution to this work, and approved the submitted version for publication.

## Funding

This work was supported by Chongqing Natural Science Foundation(cstc2019jcyj-msxmX0173).

## Conflict of Interest

The authors declare that the research was conducted in the absence of any commercial or financial relationships that could be construed as a potential conflict of interest.
